# Dynamic Link between Histone H3 Acetylation and an Increase in the Functional Characteristics of Human ESC/iPSC-Derived Cardiomyocytes

**DOI:** 10.1371/journal.pone.0045010

**Published:** 2012-09-12

**Authors:** Tomomi G. Otsuji, Yuko Kurose, Hirofumi Suemori, Masako Tada, Norio Nakatsuji

**Affiliations:** 1 Stem Cell and Drug Discovery Institute, Kyoto, Japan; 2 Institute for Frontier Medical Sciences, Kyoto University, Kawahara-cho, Sakyo-ku, Kyoto, Japan; 3 Institute for Integrated Cell-Material Sciences, Kyoto University, Ushinomiya-cho, Yoshida, Sakyo-ku, Kyoto, Japan; 4 Chromosome Engineering Research Center, Tottori University, Yonago, Tottori, Japan; University of Minnesota, United States of America

## Abstract

Cardiomyocytes (CMs) derived from human embryonic stem cells (hESCs) or human induced pluripotent stem cells (hiPSCs) are functionally heterogeneous, display insufficient biological efficacy and generally possess the electrophysiological properties seen in fetal CMs. However, a homogenous population of hESC/hiPSC-CMs, with properties similar to those of adult human ventricular cells, is required for use in drug cardiotoxicity screening. Unfortunately, despite the requirement for the functional characteristics of post-mitotic beating cell aggregates to mimic the behavior of mature cardiomyocytes *in vitro*, few technological improvements have been made in this field to date. Previously, we showed that culturing hESC-CMs under low-adhesion conditions with cyclic replating confers continuous contractility on the cells, leading to a functional increase in cardiac gene expression and electrophysiological properties over time. The current study reveals that culturing hESC/hiPSC-CMs under non-adhesive culture conditions enhances the electrophysiological properties of the CMs through an increase in the acetylation of histone H3 lysine residues, as confirmed by western blot analyses. Histone H3 acetylation was induced chemically by treating primitive hESC/hiPSC-CMs with Trichostatin A (TSA), a histone deacetylase (HDAC) inhibitor, resulting in an immediate increase in global cardiac gene expression. In functional analyses using multi-electrode array (MEA) recordings, TSA-treated hESC/hiPSC-CM colonies showed appropriate responses to particular concentrations of known potassium ion channel inhibitors. Thus, the combination of a cell-autonomous functional increase in response to non-adhesive culture and short-term TSA treatment of hESC/hiPSC-CM colonies cultured on MEA electrodes will help to make cardiac toxicity tests more accurate and reproducible via genome-wide chromatin activation.

## Introduction

Human embryonic stem cells (ESCs) are pluripotent stem cells derived from the inner cell mass of the blastocyst, whereas induced pluripotent stem cells (iPSCs) are a type of pluripotent stem cell artificially derived from reprogrammed somatic cells [1], [2]. Both cell types can be used to generate vast numbers of cells, which can then develop into human tissues. In particular, hESC/hiPSCs are currently regarded as the most promising source of cardiomyocytes for use in drug discovery and drug safety pharmacology [3], [4]. Efficient methods of producing cardiomyocytes from hESC/hiPSCs have been developed and improved [5], [6], [7]; however, hESC-derived cardiomyocytes (hESC-CMs) are immature compared with adult human cardiomyocytes, and this has recently become a topic of discussion [8], [9], [10], [11]. Cardiac cells diverge functionally during embryonic development, and after the mitotic arrest that occurs in mammalian cardiomyocytes early in postnatal development, each cell continues to grow to produce a heart of the appropriate size. Concurrently, each cell increases the function of a set of ion channels during embryonic and postnatal development [5], [12], [13], [14]. Ion channel gene expression profiles reflect CM characteristics and show variation between immature and mature CMs. Early gene expression patterns lead to weak cell-autonomous contractility even in ventricular CMs; however, later in development, adult ventricular cells lose their cell-autonomous contractility and mature cardiomyocytes contract in response to impulses generated by pacemaker cells, which mature coordinately. Most reports state that hESC-CMs/hiPSC-CMs show autonomous contractility, indicating their immaturity. In *in vitro* cardiac toxicity tests with hESC-CMs/hiPSC-CMs, the developmental stage of the cells used is very important; however, the mechanisms involved in age-related functional development in post-mitotic cardiomyocytes are still uncertain.

Automaticity declines rapidly in hESC-CM aggregates during adhesive culture. However, this is not due to the increasing maturity of ventricular cells but to early loss of the pacemaker cell lineage in the aggregates [15], [16]. For the current study, we succeeded in maintaining the contractility of hESC-CM aggregates over a year by periodically replating the beating CM spheroids every 2 weeks. In addition, the functional characteristics of 8-month-old hESC-CMs were demonstrated using multi-electrode array (MEA), patch-clamp and quantitative RT-PCR (qRT-PCR) analyses, in which cardiac gene expression, ion current amplitudes and dose-dependent responses to the human Ether-a-go-go Related Gene (hERG) ion channel blockades were increased [17]. Moreover, we found that non-adhesive culture (three-dimensional culture (3D)), for at least 2 weeks, restored the global gene repressive status that had been established during adhesive culture. Finally, it was possible to maintain beating hESC-CM spheroids that behaved as a functional syncytium, with ventricular cells and a pacemaker cell mass, after long-term, low-adhesive culture. However, low-adhesive culture is time-consuming; therefore, another culture method in which the cells mature more quickly is required.

In general, appropriate chromatin regulation is necessary for the correct development of cells within a particular tissue. Increased acetylation of N-terminal lysine residues of histones H3 and H4 by histone acetylases (HATs) correlates with increased transcription as the folded chromatin becomes more accessible to the transcriptional machinery. By contrast, histone deacetylases (HDACs) remove the acetyl groups from the lysine residues, resulting in condensed and transcriptionally silent chromatin [18]. The aim of this study was to generate a homogeneous population of cardiomyocytes with functional characteristics similar to those of adult cardiomyocytes for cardiac toxicity tests. Thus, we expected that low-adhesive culture might increase histone acetylation levels and electrophysiological function in hESC/hiPSC-CMs. In this study, 3D-cultured hESC/hiPSC-CMs showed higher acetylation levels, as demonstrated by western blotting. Moreover, Trichostatin A (TSA)-induced histone acetylation activated transcription in general, and in particular, the expression of a set of ion channel genes in the hESC/hiPSC-CMs. Short-term TSA treatment of hESC/hiPSC-CMs cultured on the probes of an MEA system dramatically improved the considerable qualitative heterogeneity seen in untreated CM spheroids in the response to hERG ion channel blockade, which is associated with life-threatening arrhythmias. Thus, important issues, such as reproducibility and scalability, which prevent the use of hESC/hiPSC-CM spheroids in cell-based drug safety assays might be largely resolved by a combination of short-term 3D culturing and simple pretreatment with HDAC inhibitors.

## Results

### Increase in Cardiac Gene Expression in hiPSC-CMs after 3D Culture

One representative iPSC line suitable for cardiac differentiation was selected from five human iPSC cell lines (253G1, 201B7, IMR90 C1, IMR90 C4 and foreskin C1) using a hESC-END-2 coculturing system. END-2 cells are a visceral endoderm-like cell line derived from mouse P19 embryonal carcinoma cells. The number of beating colonies on Day 21 after co-culture varied among these hiPSC lines; however, the 253G1 and 201B7 lines produced about six-fold more beating colonies than the well-characterized human ES cell line, KhES-1 [17] (Fig. S1A).

Next, qualitative RT-PCR (qRT-PCR) analysis was used to compare expression levels of the cardiac genes, alpha myosin heavy chain (αMHC), ERG1b and KCNQ1, in the five hiPSC-CMs with the levels in the hESC-CM line, KhES-1. Gene expression levels in cardiomyocytes derived from the 253G1 and 207B7 lines, were comparable to those in the hESC-CM line, KhES-1 (Fig. S1B). These data indicated that 253G1 and 201B7 were more efficient than the other hiPSC lines in producing cardiac cells in the END-2 co-culture system. Accordingly, 253G1 hiPSCs and KhES-l hESCs were used for the remainder of the study. Next, we investigated whether 3D culture could enhance cardiac gene expression, not only in hESC-CMs but also in hiPSC-CMs. Primary hiPSC-CMs (21 days-old) were cultured either in adhesion (Ad) or in suspension (Sus) culture for 14 days. The 3D culture system is sometimes referred to as 'Sus' to avoid confusion with 3-day culture. The qRT-PCR analysis revealed that the gene expression levels of αMHC, ERG1b and KCNQ1 were higher after 14 days of Sus culture than after the same period of Ad culture (Fig. S1C).

### Free Contractility Increases Histone H3 Acetylation and Cardiac Gene Expression in hESC-CMs

**Figure 1:**
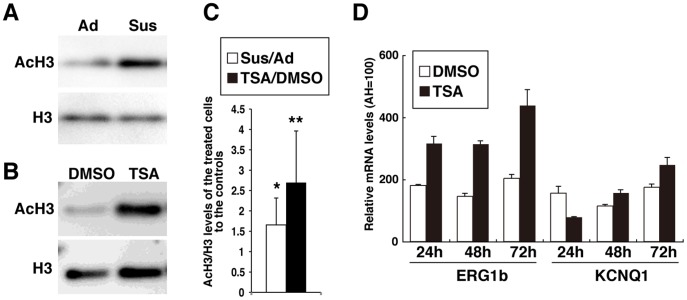
Effect of suspension culture and TSA treatment on histone acetylation of hESC-CM colonies. Western blot analyses show increased histone H3 acetylation (AcH3) levels in 35-day-old hESC-CMs (A) following 14 days of suspension culture (Sus) or (B) following treatment with 100 nM TSA for 48 hrs under adhesive (Ad) culture conditions (DMSO treatment had no effect on AcH3 levels). (C) Results from western blot analyses comparing Sus to Ad cultures (n = 9) and TSA to DMSO treatments (n = 13) show increased AcH3 levels in hESC-CMs. (D) Results of qRT-PCR analysis showing that TSA increases cardiac gene expression in hESC-CMs for at least 48 hrs after treatment. The corresponding values for human adult hearts (AH) are shown for each sample. Each graph displays the mean and standard deviation (SD) of three independent experiments. *, p<0.05; **, p<0.01.

Next, the question of how 3D culture enhances cardiac gene expression and function was addressed. In general, histone acetylation-mediated chromatin activation is associated with enhanced gene expression; therefore, histone H3 acetylation levels (AcH3) were examined in hESC-CMs by western blot analysis. Three sets of comparative 14-day cell cultures were prepared for western blot analyses to compare AcH3 levels directly between Sus-cultured samples and Ad-cultured controls. Moreover, to help with the precise quantification of AcH3 levels, western blot analyses were performed three times for each sample. In total, nine western blots comparing Sus to Ad cultures showed that H3 acetylation levels were 1.7±0.6 times greater in Sus cultures than in Ad cultures (Fig. 1A and 1C). Positive effect induced by Sus culturing on the increase of AcH3 level was statistically significant in hESC-CMs (p<0.05). Thus, 3D culture seems to activate the transcription of genes related to mature hESC-CM phenotypes through *de novo* histone acetylation. Global histone acetylation was then induced indirectly using TSA, an HDAC inhibitor, under adhesive culture conditions. TSA is a selective inhibitor of the class I and II mammalian HDAC families and has emerged as a promising epigenetic drug for various disorders caused by deregulation of the equilibrium between HATs and HDACs [19]. TSA was compared with dimethyl sulfoxide (DMSO), the solvent for TSA and, as expected, 100 nM TSA was more efficient at inducing acetylation of H3 lysine residues in hESC-CMs under adhesive culture conditions (Fig. 1B). Five sets of comparative cell cultures were prepared for western blot analyses to compare AcH3 levels directly between TSA-treated samples and DMSO-treated controls. With western blotting performed in double or triplicate for each sample, the comparison between TSA and DMSO was performed a total of 13 times and the results showed that H3 acetylation levels were 2.7±1.3 times greater in TSA-treated hESC-CMs than in DMSO-treated controls (Fig. 1C). The effect of TSA on the increase in AcH3 level in hESC-CMs was statistically significant (p<0.01). Thus, for the current study, TSA was used as a reliable chemical for inducing histone acetylation.

**Figure 2:**
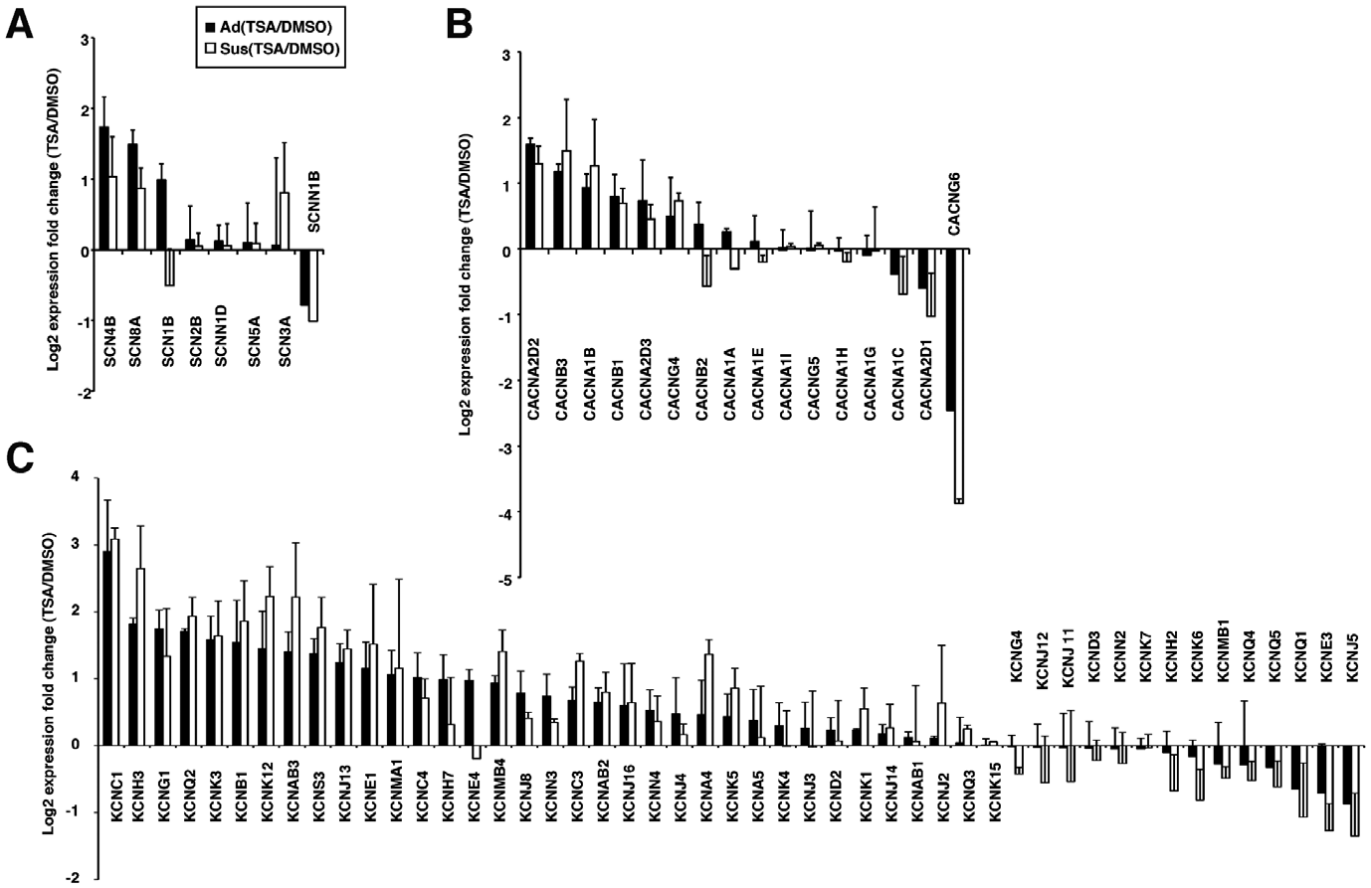
Microarray data for the expression of genes involved in cardiac ion channel function. Global gene expression analyses were conducted using TSA- or DMSO-treated hESC-CMs under the Ad culture conditions (black bar) or Sus culture conditions (white bar). Genes involved in (A) sodium, (B) calcium and (C) potassium ion channels were analyzed. Each graph displays the mean and SD of two or three independent experiments.

ERG1b and KCNQ1 gene expression increased at 48 and 72 hrs in TSA-treated hESC-CMs compared with DMSO-treated cells, as detected by qRT-PCR (Fig. 1D). Three sets of microarray analyses comparing TSA-treated hESC-CMs with DMSO-treated controls were analyzed under Ad culture conditions, described as Ad(TSA/DMSO) (Fig. 2 and Fig. S2). In addition, two sets of comparative analyses for TSA and DMSO were performed under Sus culture conditions, described as Sus(TSA/DMSO). Following TSA pretreatment, the expression levels of internal control genes for comparative gene expression analyses, such as β-actin and RPL13A, but not GAPDH, maintained stable (Fig. S2). Thus, β-actin was selected as a control gene for subsequent expression analyses. The results showed that TSA induced concerted changes in gene expression profiles in both Ad and Sus cultures. Although we found no link between altered gene expression profiles and enhanced function, TSA enhanced the gene expression of two of eight sodium ion channel-related genes, two of 16 calcium ion channel-related genes, and 16 of 49 potassium ion channel-related genes by more than two-fold in hESC-CMs, but repressed some of the remaining genes (Fig. 2 and Fig. S2). From these data, it was expected that TSA might enhance the electrophysiological function of hESC-CMs under adhesive culture conditions without long-term contractile culture.

### Early hESC-CM Colonies Show Heterogeneous Responses to the HERG Inhibitor, E4031, in MEA Tests

Electrophysiological function was analyzed using an MEA test in beating cell aggregates of 35-day-old hESC-CMs after 2 weeks of 3D culture. MEA measures the extracellular field potential (FP) produced by the electrical activity of many cells, and the cardiac cell FP duration (FPD) on MEAs has been shown to correspond to QT interval properties seen in an electrocardiogram (ECG) *in vivo.* Thus, the advantage of MEA testing is that the results are easier to understand and show the electrophysiological function of the beating cell units with minimum of intercellular functional heterogeneity. During the electrophysiological tests, the cardiomyocytes need to be attached tightly to the electrode array in the chamber; therefore, 2 days of adhesive culture on the electrodes were required to record FPs.

**Figure 3:**
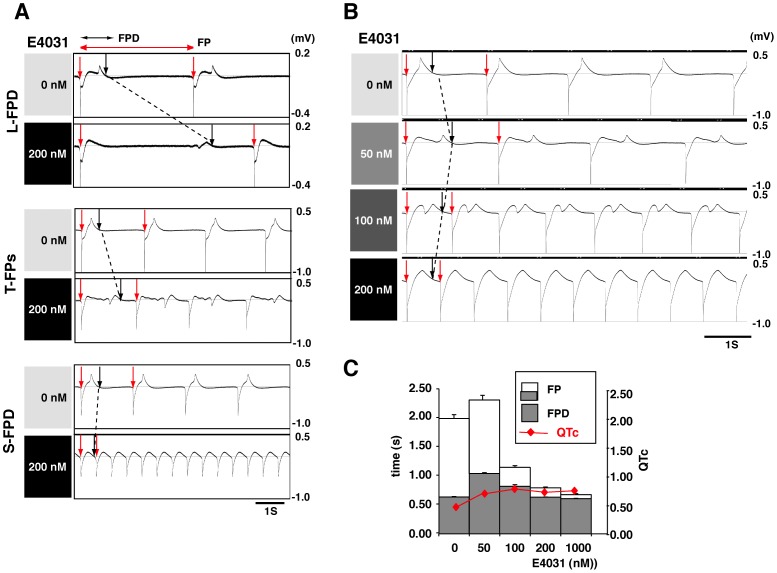
Heterogeneous responses to the hERG inhibitor, E4031, in MEA tests using early hESC/hiPSC-CM colonies. (A) Sensitivity to 200 nM E4031 was measured in Sus cultured hiPSC-CM colonies (d35) after an additional 2 days of Ad culture on multi-electrode array (MEA) probes. The hiPSC-CM colonies showed the following heterogeneous responses to the hERG inhibitor: lengthened field potential duration (L-FPD), shortened FPD (S-FPD), and transient FP phenotypes (T-FPs). Red arrows indicate the initiation of FPs and the black arrows show the end-point of the cardiac repolarization phase in the FP signals. The broken line shows the shift in depolarization from 0 nM through 200 nM E4031. (B, C) Dose increment analyses in Sus-cultured (d35) hESC-CM colonies. The colony showing the L-FPD response to 50 nM of E4031 showed an S-FPD response at 200 nM through to a T-FPs response at 100 nM. This indicates that some of the Sus-cultured (d35) hESC-CM colonies were more sensitive to E4031 than others. However, on the basis of the rate-corrected QT (QTc) values, the S-FPD spheroids also show QTc prolongation (red line), because of the cells’ shortened FP rates. The whole bar containing a gray part displays the mean and SD of six FP cycles, and the gray bar displays the mean and SD of FPD.

Even when 3D culture enhanced gene expression levels in immature hESC/iPSC-CM spheroids, variable responses to hERG channel inhibition by E4031 were clearly observed. The 200 nM E4031-treated hESC/iPSC-CM spheroids showed complex responses with regard to changes in FP morphologies, the spontaneous FP rates, and the FPD time. The responses were categorized into four groups for descriptive purposes: lengthened FPD (L-FPD), shortened FPD (S-FPD), transient FP phenotypes (T-FPs), and loss of contractility (LOC) (Fig. 3A). The CMs involved in the LOC group lost automaticity with E4031 treatment, resulting in an absence of MEA signals. The T-FPs described a transient electrophysiological phenotype that ranged from L-FPD to S-FPD in response to a dose increment assay with I_Kr_ inhibitors; however, the mechanism involved is unclear. Some early hESC-CM spheroids showed a phenotypic shift from L-FPD detected at 50 nM, through T-FPs at 100 nM, to S-FPD at 200 nM (Fig. 3B). At least for the L-FPD and S-FPD groups, the major difference observed was in the FP rates, and the FPD time between the beginning of depolarization and the end of repolarization needed to be adjusted for these rate variations. Thus, the QTc correction was used to control differences in rate. When the rate correction was applied, not only L-FPD but also S-FPD and T-FPs groups tested showed an increase in calculated QTc (Fig. 3C).

Changes in electrophysiological phenotypes from L-FPD through T-FPs to S-FPD are likely to reflect a developmental shift in target molecules for E4031 exposed on the hESC-CM cell surface. This hypothesis is supported by our previous MEA tests using 8-month-old hESC-CMs that reproducibly showed an L-FPD phenotype in response to 200 nM of E4031 [17]. Moreover, our previous patch-clump analyses also showed that depolarization was mostly initiated by an inward Na^+^ current in the 8-month-old hESC-CMs, while immature hESC-CMs showed self-contractility initiated by insufficient depolarization, induced mainly by inward Ca^2+^ current. Therefore, although the major depolarization current in hESC-CMs was uncertain in the current MEA tests, the data suggest that early hESC-CM colonies are still developmentally heterogeneous, leading to the frequent detection of T-FPs and S-FPD electrophysiological phenotypes. Thus, even if 3D culture temporally increases gene expression levels in immature hESC-CMs, this positive effect seems to disappear following adhesive culture during MEA testing.

### HDAC Inhibitor Improves MEA Testing in hESC-CMs

Next, we examined the effects of TSA-mediated acetylation on the MEA testing of a transgenic hESC cell line expressing enhanced green fluorescent protein (EGFP) under the control of the αMHC promoter. The hESC-CM colonies were cultured for 21 days under adhesive conditions, and then the contracting αMHC-EGFP hESC-CM colonies were cultured in suspension for 2 to 4 weeks before plating on electrodes for MEA recordings. MEA testing was performed sequentially on Day 2, Day 6 and Day 9 after the hESC-CM colonies were placed on the electrodes.

**Figure 4:**
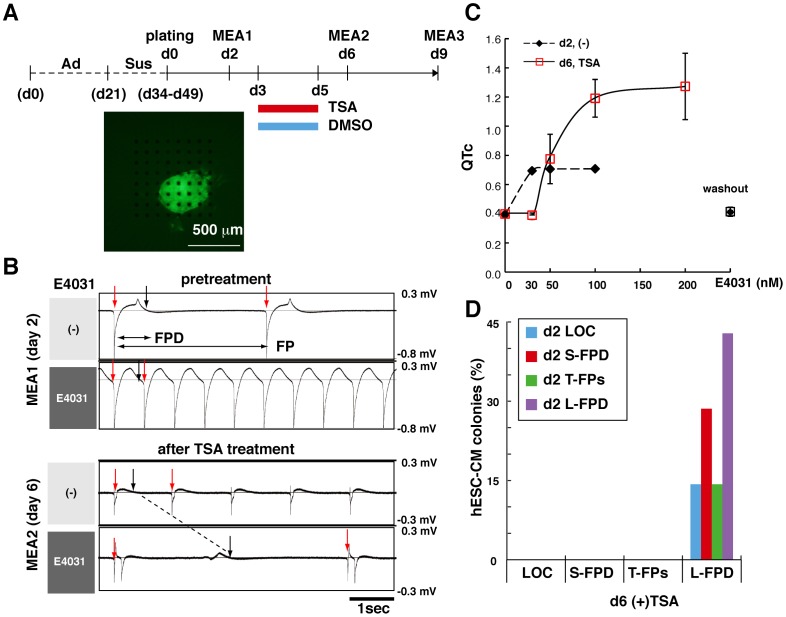
TSA enhances electrophysiological function in hESC-CMs. (A) Experimental scheme: 21-day-old beating colonies obtained from αMHC-EGFP transgenic hESCs were maintained for 13 to 28 days in suspension. Sensitivity to 200 nM E4031 was measured three times for each beating colony using an MEA system on Days 2, 6, and 9 after the colonies were plated on the MEA probes, as shown at the lower panel. DMSO, or 100 nM TSA, was added for 48 hrs between Days 3 and 5 and then removed for 1 day before the second MEA recording to avoid contact with ion channel inhibitors. (B) Sensitivity of EGFP-positive hESC-CMs to E4031 before TSA treatment (Day 2) and after TSA treatment (Day 6) was measured using an MEA system. Representative FPs were demonstrated in hESC-CM spheroids before and after 100 nM E4031 treatment showing phenotypic changes in the response to 100 nM E4031 from S-FPD to L-FPD in TSA-treated hESC-CM spheroids. (C) Dose-dependent QTc prolongation seen in TSA-treated hESC-CM colonies that had shown arrhythmia in response to 100 nM E4031 in the first MEA test. Each value displays the mean and SD of FPDs obtained from six contiguous FP cycles. (D) Sequential MEA analyses of every hESC-CM colony before and after TSA treatment showing that all colonies reproducibly acquired a uniform electrophysiological phenotype with 2 days of TSA treatment (n = 7).

To analyze the effects of TSA on sensitivity to the hERG inhibitor, E4031, each plated colony was treated with either TSA or DMSO for 48 hrs starting 1 day after the first MEA test (Fig. 4A). On Day 2, 45% of the hESC-CM colonies showed an L-FPD phenotype in response to 200 nM E4031. The remaining immature CM colonies showed T-FPs, S-FPD or LOC phenotypes (Fig. 4B, upper panel), followed by a qualitative change in phenotype from the arrhythmic S-FPD type to L-FPD after TSA treatment (Fig. 4B, lower panel). In addition, some immature CM spheroids showed S-FPD to E4031 on Day 2 after plating on the MEA electrodes, but exhibited dose-dependent FPD prolongation on Day 6 after TSA treatment (Fig. 4C). This type of spheroid contributed to the Day 2 S-FPD and Day 6 (+) TSA L-FPD groups, as shown in Figure 4D. Moreover, all of the hESC-CM colonies treated with 100 nM TSA showed an L-FPD phenotype on Day 6, 4 days after the first MEA test (Fig. 4D). On the other hand, only 17% of the DMSO-treated hESC-CM colonies showed an L-FPD phenotype on Day 6.

**Figure 5:**
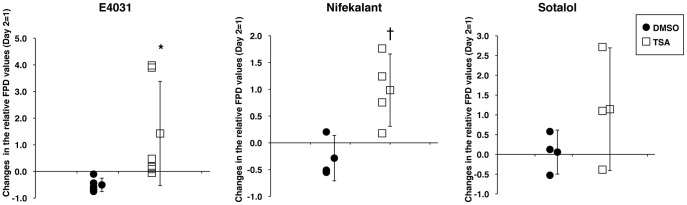
Responses to the ion channel blockers, E4031, Nifekalant and Sotalol, are improved in TSA-treated hESC-CMs. Relative FPD values were obtained with the MEA system on Day 2 to Day 6 for each colony in the presence of ion channel inhibitor (200 nM E4031; 20 µM Nifekalant; 200 µM Sotalol). Each graph displays changes in the relative FPD values in each hESC-CM colony after DMSO or TSA treatment and the mean and SD (E4031, n = 5 for DMSO and n = 6 for TSA; Nifekalant, n = 3 for DMSO and n = 4 for TSA; Sotalol, n = 3 for DMSO and n = 3 for TSA). *, p<0.05; †, tendency to differ (p<0.1).

Cardiac FP cycles were sequentially recorded and the average FPD of six cycles in the presence of 200 nM E4031 was calculated for each hESC-CM and hiPSC-CM colony. The average FPD recorded on Day 6 or Day 9 was normalized to that of Day 2 for each colony. Changes in relative FPD were then evaluated for DMSO or TSA treatment. The DMSO-treated hESC-CM colonies remained essentially unchanged from Day 2 to Day 6, with a difference in the relative FPD values of −0.5±0.25 (n = 5). By contrarast, the average FPD values more than doubled in the hESC-CM colonies after TSA treatment, with a difference in relative FPD values of 1.43±1.95 (n = 6) as shown in Figure 5. The effect of TSA on the extended prolongation of FPD was statistically significant in hESC-CMs treated with 200 nM E4031 treatment (p<0.05). Two other potassium ion channel inhibitors, Nifekalant and *dl*-Sotalol, both of which induce FPD prolongation were also applied to TSA-treated hESC-CMs; however, it is worth noting that *dl*-Sotalol is known to trigger false negative responses in *in vitro* hERG assays [20]. FPD prolongation was pronounced approximately doubled following treatment with 20 µM Nifekalant treatment {DMSO, −0.28±0.42 (n = 3); TSA, 0.98±0.68 (n = 4)} or 200 µM *dl*-Sotalol treatment {DMSO, 0.06±0.56 (n = 3); TSA, 1.14±1.55 (n = 3)} in TSA-treated hESC-CMs, but not in DMSO-treated hESC-CMs (Fig. 5). The tendency for different effects of TSA and DMSO on the extended prolongation of FPD was demonstrated for Nifekarant (p<0.1); however, the effect of TSA for *dl*-Sotalol was not statistically significant.

### Positive Effect of TSA Treatment on MEA Testing in hESC-CMs is Transient

**Figure 6:**
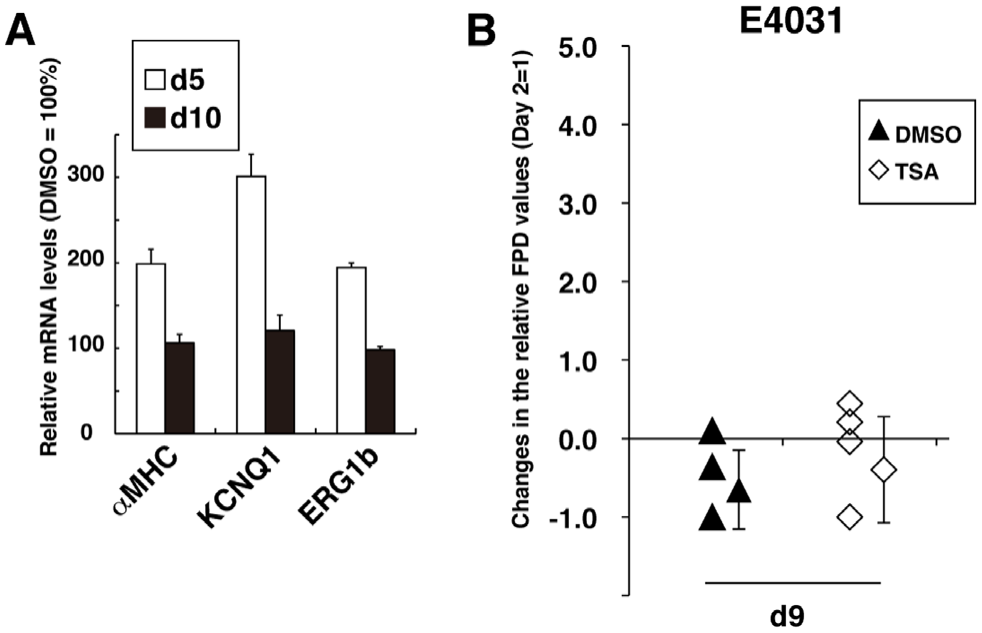
The TSA-mediated positive effect on MEA testing is transient. (A) Results of qRT-PCR analyses of cardiac gene expression in hESC-CMs showed that the effect of TSA was transient. Day 5 (d5): immediately after 2 days of TSA treatment; Day 10 (d10): 5 days of culture after TSA washout. Values relative to the DMSO-treated hESC-CMs (DMSO = 100%) are shown (n = 3). Values represent the mean ± SD for each set of measurements. (B) Sequential MEA analyses of all hESC-CM colonies were performed on Days 2, 6, and 9 in the presence of 200 nM E4031. Changes in the relative FPD values on Day 6 or Day 9 from Day 2 for each colony are shown in Figures 5 and 6B, respectively. Values represent the mean ± SD (n = 5 for DMSO, n = 6 for TSA). Significant differences between DMSO and TSA treatment detected on Day 6 were not detected on Day 9.

The qRT-PCR analyses revealed that, after washing out TSA, the expression levels of αMHC, KCNQ1 and ERG1b were reduced to the levels seen in DMSO-treated hESC-CMs within 5 days (Fig. 6A, d10). Spontaneous beating rates in Day 2 hESC-CM colonies before TSA treatment were approximately 21.1±5.4 beats per min (bpm) (Fig. S3A), which is slower than the beating rate of an adult or neonatal heart. Interestingly, the beating rate increased to 34.0±7.5 bmp on Day 6 in TSA-treated hESC-CM colonies, but not in DMSO-treated CM colonies. However, this TSA-mediated increase in beating rate was likewise extinguished within 3 days after TSA washout (Fig. S3A). FPD prolongation, induced by 200 nM E4031, was enhanced significantly in TSA-treated hESC-CM colonies compared with DMSO-treated controls on Day 6 (Fig. 5). The positive effect of TSA on the MEA-mediated cardiac toxicity test was also found to be transient, as the TSA-treated colonies and the DMSO-treated hESC-CM colonies responded similarly to E4031 on Day 9 after plating onto MEA probes {DMSO, −0.65±0.50 (n = 5); TSA, −0.40±0.68 (n = 6)} as shown in Figure 6B.

### Effects of HDAC Inhibitor on MEA Testing in hiPSC-CMs

**Figure 7:**
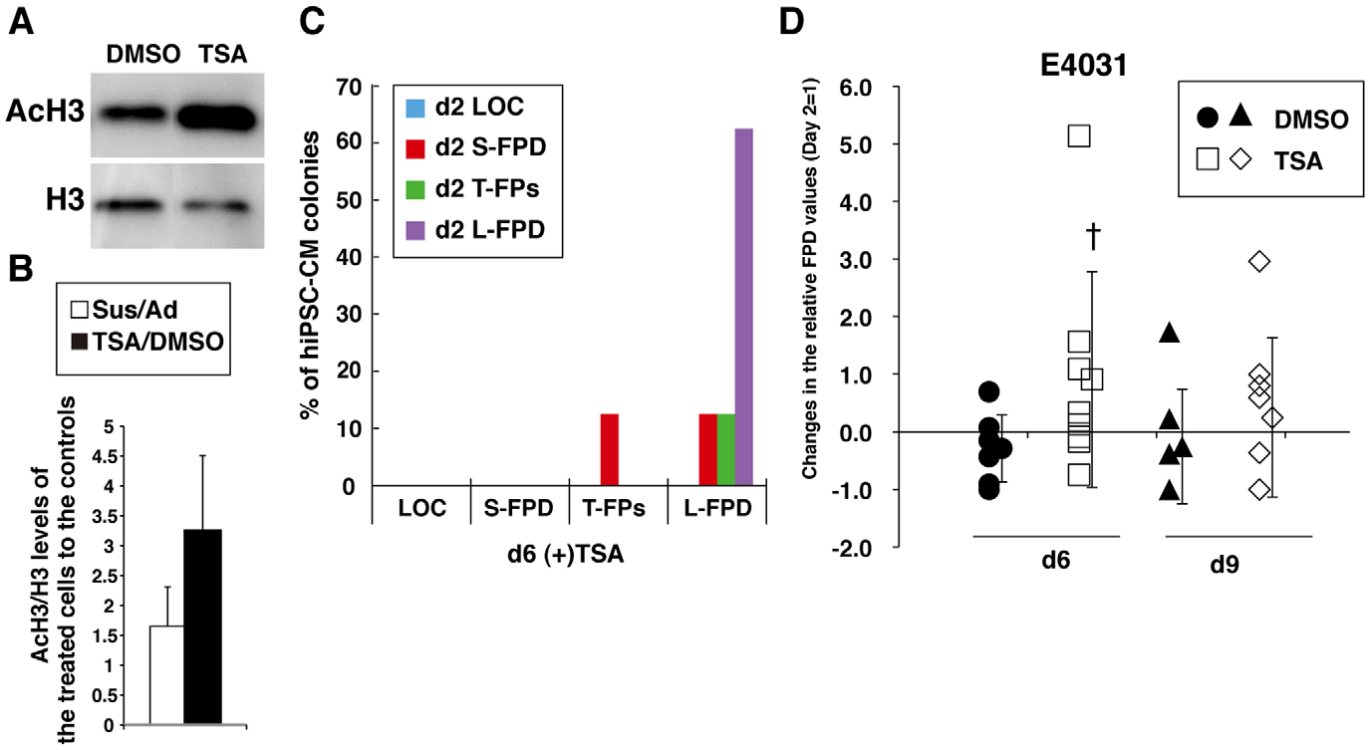
TSA enhances electrophysiological function in hiPSC-CMs accompanied by histone H3 acetylation. (A) Western blot analyses show increased histone H3 acetylation levels in hiPSC-CMs treated with 100 nM TSA for 48 hrs. (B) Results from western blot analyses comparing Sus to Ad cultures (n = 7) and TSA to DMSO treatments (n = 9) show increased AcH3 levels in hiPSC-CMs. (C) Sequential MEA analyses in all hiPSC-CM colonies on Day 2 and Day 6 showed that TSA altered the electrophysiological phenotypes evaluated on the basis of their sensitivity to 200 nM of E4031 (n = 8). (D) Sequential MEA analyses in all hiPSC-CM spheroid on Days 2, 6 and 9 after plating showed electrophysiological phenotypes improved reproducibly following 2 days of TSA treatment in the presence of 200 nM E4031 (n = 7 for DMSO, n = 8 for TSA). Changes in relative FPD values (Day 2 = 1) from Day 2 to Days 6 and 9 and the mean±SD are shown. †, tendency to differ (p<0.1) only on Day 6.

Finally, to confirm whether TSA enhances cardiac function in hiPSC-CMs as well as in hESC-CMs, the hiPSC-derived primitive CMs (cell line 253G1) were treated with TSA. Western blot analysis was performed seven times to compare the Sus and Ad culture techniques for three sets of comparative cultures, and nine times to compare the effects of TSA and DMSO for three sets of comparative cultures in hiPSC-CMs. The results showed that Sus culture and TSA increased the histone H3 acetylation levels 1.7±1.3 and 3.3±2.8 times, respectively, over their respective controls, (Fig. 7A and B). However, the effect of TSA on the increase of AcH3 level in hiPSC-CMs was not statistically significant. TSA also increased the beating rates over that observed in DMSO-treated hiPSC-CMs on Day 6 (Fig. S3B). In addition, TSA treatment increased the frequency of L-FPD spheroids from 63% on Day 2 to 88% on Day 6 (Fig. 7C). Moreover, the FPDs in TSA-treated hiPSC-CMs doubled in response to 200 nM E4031 (Fig. 7D). Day 6 MEA measurements demonstrated enhanced FPD elongation in TSA-treated hiPSC-CM spheroids {DMSO, −0.29±0.99 (n = 7); TSA, 0.91±1.87 (n = 8)}, although the tendency for different effects of TSA and DMSO on the extended prolongation of FPD was only shown for E4031 (p<0.1) in ihPSC-CMs. These effects were again eradicated 3 days later on Day 9 {DMSO, −0.29±0.58 (n = 7); TSA, 0.25±1.38 (n = 8)} (Fig. 7D and Fig. S3B). Thus, we concluded that appropriate chromatin regulation with the aid of TSA could temporarily enhance expression of a set of cardiac genes, and will help in the preparation of functionally uniform hESC/hiPSC-CM colonies and enable reliable MEA-mediated cardiac toxicity tests.

## Discussion

Cardiac function increases during embryonic development and postnatal growth, and this increase is associated with changes in ionic currents and cardiac action potentials. There are several different types of ionic current involved in action potential repolarization, and these can be species-, tissue- and even age-specific. In addition, the immature and mature heart differs in terms of excitability, action potential properties, contractility and gene expression profiles [12], [21]. On the basis of experimental animal studies, it is suggested that the immature mammalian myocardium is more sensitive to the action potential duration- (APD-) prolonging effects of I_Kr_ blockades than the adult myocardium [13]. In this study, we demonstrated that primitive hESC/hiPSC-CMs were often sensitive to the hERG K^+^ channel inhibitor, E4031, even at low concentrations, which may result in the loss of availability of potentially useful chemicals due to false positive results from cardiac toxicity tests during the drug screening process. Thus, hESC/hiPSC-CMs with homogeneous properties similar to adult human ventricular cells are required for accurate drug cardiotoxicity screening.

In mice, mitochondrial staining with the MitoTracker dye, tetramethylrhodamine methyl ester perchlorate (TMRM), revealed that an abundance of cellular mitochondria is a common characteristic of cardiomyocytes, but not of cardiac progenitor cells, and suggested that primary cardiac differentiation can be achieved during an initial 2 week differentiation period [22]. However, patch-clamp analyses revealed that 2-week-old primitive hESC/hiPSC-CMs are functionally insufficient. The system of co-culture of hESCs with mouse END-2 cells as described in the current study is a very efficient method for the induction of a ventricular-like cell lineage [23]; however, developmentally, these cells are comparable to 16-week-old fetal ventricular CMs *in vivo* [8].

Recently, we reported a method for producing primitive hESC-CM spheroids with more mature electrophysiological phenotypes by cyclic replating onto a new culture dish every 2 weeks [17]. This low-adhesive culture technique generates cells that are capable of maintaining free contractility for over a year, resulting in significant increments of the inward sodium current I_Na_, the inward calcium current I_Ca_, the inward potassium rectifier current I_K1_, and the rapid (I_Kr_) and slow (I_Ks_) components of the delayed potassium rectifier current in 8-month-old ventricular-like hESC-CMs, as well as the pacemaker current (If) in long-term cultured pacemaker-like CMs, accompanied by global up-regulation of cardiac gene expression. In the MEA assay combined with hESC-CMs, dose-dependent FPD prolongation for hERG inhibitors was detected reproducibly after 3 months of low-adhesive culture. When L-FPD spheroids are used in the MEA assay, FPD prolongation is obvious and the QTc evaluation is not necessary. In the current study, 1- to 2-month-old hiPSC-CMs also showed high sensitivity to low concentrations of hERG inhibitors as seen in immature hESC-CMs, even though gene expression levels were increased following the addition of a 2 week period of 3D culture. In MEA recordings, functional reduction, induced by adhesive culturing on electrodes, was a fundamental issue that could not be avoided, especially in immature CMs.

The gene expression profiles of many genes are regulated epigenetically. For example, HATs and HDACs alter chromatin structure, which affects gene transcription; HATs facilitate chromatin opening by acetylating the N-terminal tails of nucleosomal histones at lysine residues. By contrast, HDACs remove the acetyl groups and hypo-acetylated lysine residues are often associated with inactive chromatin [24]. In this study, we showed that 3D culture increases global histone H3 acetylation in hESC/hiPSC-CMs. Moreover, the histone deacetylase inhibitor, TSA, also increased histone H3 acetylation of hESC/hiPSC-CMs under adhesive culture conditions within 2 days of treatment, and promoted a qualitative shift from immature CMs that were hypersensitive to three I_Kr_ inhibitors, E4031, Nifekalant and Sotalol, towards CMs showing obvious FPD prolongation. However, following washing out, TSA treatment no longer effectively elevated electrophysiological function, suggesting that both 2-week 3D culture, and 2-day TSA treatment under adhesive conditions, temporarily enhanced hESC/hiPSC-CM function by increasing global gene expression through protein acetylation, while extended short-duration adhesive culture induced global gene repression through histone deacetylation.

Previously, it was shown that cyclic mechanical stretch (CMS) is an important physiological and pathological factor in the heart: CMS, induced experimentally for 24 hours in 7-day cultured neonatal rat cardiomyocytes, induced elongation in the direction of the force and changed the distribution of Connexin 43 (Cx43) and N-Cadherin as well as inducing a significant increase in Cx43, certain transcription factors and the phosphorylated forms of ERK1/2 [25]. Interestingly, the CMS-induced changes in Cx43 were reversible within 24 hours of removal of the force.

There is further evidence showing that cell adhesion status regulates histone acetylation through intracellular contractility-related signaling, not only in cardiac cells, but also in gastric carcinoma cells [26]. Gastric carcinoma cells in suspension show higher levels of histone acetylation compared with fibronectin-adherent cells, while chemical inhibition of intracellular contractility-related myosin light chain (MLC) kinase decreases basal and TSA-mediated histone H3 acetylation in suspension. These observations, along with our own results, show that the maintenance of cell contractility is closely related to cell-autonomous maturation in cardiomyocytes. Since ventricular and atrial cells become auto-quiescent once they are functionally mature, co-existence with sinoatrial nodal cells derived from ESC/hiPSCs will be a key factor in the *in vitro* cell-autonomous maturation of hESC/hiPSC-ventricular cells.

Cardiac myocytes in mammals proliferate rapidly during fetal life but exit the cell cycle soon after birth [14]. In addition, it is likely that hESC/hiPSC-CMs also exit the cell cycle after primary cardiac differentiation, because the size of beating colonies remained unchanged during long-term culture and the cells were negative for Ki67, a widely-used cell proliferation marker. Therefore, the acetylation of nucleosomal histones mediated by 3D culture and TSA treatment seems to be a *de novo* event. Currently, TSA is used as an epigenetic drug *in vivo* for heart hypertrophy disease in experimental animals [27], [28], [29]. Interestingly, TSA arrests the progress of heart hypertrophy but also reduces its advanced symptoms, indicating that the chromatin structure of cardiomyocytes seems to be partly reversible without the cells having to go through cell division [27].

A typical example of such reversible chromatin structure is seen in the developmental shift of the myosin heavy chain (MHC) isoforms of the cardiac sarcomere protein, which functions in heart contraction. Myosin is composed of two MHC and four MLC isoforms. An αMHC homodimer predominates in adult mice, while the beta MHC (βMHC) homodimer predominates in the ventricles of fetal mice. In contrast, the βMHC homodimer predominates in adult large mammals and humans [30]. In failing hearts, however, regardless of the etiology of the disease, the fast αMHC is invariably decreased with respect to the slow βMHC. In humans, this substantial reduction in αMHC expression is thought to result in a decrease in myosin ATPase enzyme velocity and a reduction in the speed of contraction [31], [32], [33].

Recently, a mechanism was put forward to explain the developmental shift from βMHC, expressed in embryos to αMHC in adult mice. In embryos, the chromatin-remodeling factor Brg1 promotes myocyte proliferation and interacts with HDAC and poly (ADP ribose) polymerase (PARP) to repress αMHC and stimulate βMHC. In adult cardiomyocytes, Brg1 is switched off but is reactivated by cardiac stress to form a complex with HDAC/PARP on the αMHC promoter, thereby inducing a pathological αMHC to βMHC shift [34]. In the present study, the increment in a proportion of αMHC to βMHC was always seen (<5% compared with the untreated controls) in TSA-treated hESC-CMs. Thus, TSA might inhibit HDAC activity involved in the repression of a set of adult-type cardiac genes thereby blunting adverse unfavorable remodeling. Moreover, although TSA increases the acetylation of GATA-4, a DNA-binding transcription factor that regulates cardiac muscle cell differentiation during heart development through activation of the cardiac-specific Nkx-2.5 promoter [35], [36], Nkx-2.5 expression was not affected in TSA-treated hESC-CMs. Moreover, our MEA tests demonstrated that the increased function of TSA-treated hESC-CMs was temporary. Thus, TSA-mediated acetylation control might enhance cardiac function but not cardiac differentiation during hESC/hiPSC-CM culturing.

To realize high-throughput drug screening and drug safety analysis using hESC/hiPSC-CMs, large quantities of uniformly mature beating colonies are required at the same time. In this study, we demonstrated that TSA was capable of producing large numbers of hESC/hiPSC-CM colonies that responded homogenously to the reference drugs in the same manner within 2 days. In addition, our results revealed that approximately 90% of the TSA-treated beating colonies showed dose-dependent FPD and QTc prolongation in response to E4031. Thus, the production of large numbers of homogeneous, mature beating colonies for use in drug testing may be possible using TSA.

## Materials and Methods

### Cardiac Differentiation of hESC/hiPSCs

Undifferentiated hESC/hiPSC colonies were cultured on mouse END-2 cells. The precise culture conditions have been described previously [17], [23]. The END-2 cells, visceral endoderm-like cells, were kindly provided by Dr. Christine L Mummery (Leiden University Medical Center, the Netherlands), which were maintained as previously described [37]. Seven established cell lines were used: two hESC lines, wild-type KhES-1 and transgenic KhES-1; and five hiPSC lines, 253G1, 201B7, IMR90 C1, IMR90 C4 and foreskin C1. The 253G1 and 201B7 lines [2], [38] were provided by the RIKEN BRC through the National Bio-Resource Project of the MEXT, Japan. The IMR90 C1, IMR90 C4 and foreskin C1 lines [39] were provided by the Wisconsin International Stem Cell Bank (WISC Bank). The hESC line, KhES1, was established and described previously by members of our research group (HS and NN) [40]. The transgenic hESC line expressing EGFP under the regulation of the αMHC promoter was established in our laboratory and has been described previously [17]. The hESC lines were used in accordance with the Guidelines for the Derivation and Utilization of Human Embryonic Stem Cells from the Ministry of Education, Culture, Sports, Science and Technology of Japan, after approval from the Institutional Review Board. On the basis of the cell’s contractility or EGFP expression of a cell, primitive hESC/hiPSC-CM colonies were removed mechanically with the tip of a micropipette on Day 21, and transferred to a new gelatin-coated dish for adhesive culture, or into 35-mm Sumilon Celltight X dishes (Sumitomo Bakelite) for 3D culture.

### Gene Expression Analyses

Total RNA isolation using an RNeasy Mini Kit (Qiagen, Hilden, Germany) and quantitative RT-PCR (qRT-PCR) were performed according to the manufacturer’s instructions. Total RNA from adult human hearts was obtained from Clontech. Briefly, cDNA was synthesized with SuperScriptIII Reverse Transcriptase, provided as part of the First-Strand Synthesis System (Invitrogen, Carlsbad, CA, USA), and was used in qRT-PCR performed using SYBR Green PCR Master Mix in a 7500 Real Time PCR System (Applied Biosystems, Foster City, CA, USA). All values were normalized with respect to β-actin expression. The primer sets used are summarized in Table S1. Each graph displays the mean and standard deviation (SD) of three independent experiments. DNA microarray analysis was outsourced to DNA Chip Research Incorporated (Japan) and was performed using the Agilent Quick Amp Labeling Kit and Agilent Human GE 4×44K Microarray (Design ID: 014850), with Agilent Feature Extraction Software, version 10.7.3.1 (Agilent Technologies, Palo Alto, CA) (n = 3 or n = 2).

### Extracellular Recordings

MEA recordings were performed as described previously [17]. Each one of the beating hESC/hiPSC-CM colonies was placed onto a gelatin-coated MEA probe (MED-P2105 or P210A, Alpha Med Scientific, Osaka, Japan) after 21 days of primary differentiation followed by 3D culture. The first extracellular recordings were performed 2 days after the CM colonies were placed on the MEA probes (Day 2) to allow time for the colonies to attach. Cells were treated with 0.002% DMSO or 100 nM TSA (Sigma-Aldrich, St. Louis, MO, USA) for 48 hours after the first MEA recordings, and then were washed twice with culture medium. The second and third recordings were performed continuously on Days 6 and 9, respectively. QTc was determined according to Bazett’s formula (QTc = FPD/√FP). An ion channel inhibitor stock solution was prepared, which contained 1 mM E4031 in H_2_O (Sigma-Aldrich), 20 mM Nifekalant hydrochloride (Wako Pure Chemical Industries), and 50 mM sotalol hydrochloride (Sigma-Aldrich) in H_2_O. Each stock solution was stored at −20°C until use.

### Western Blot Analyses

More than three sets of CMs cultured under comparative conditions were prepared. The hESC/hiPSC-CM colonies were homogenized with phosphate buffered saline (PBS) containing 1% SDS. Proteins were separated by electrophoresis on 15% SDS-polyacrylamide gels and transferred onto polyvinylidene fluoride (PVDF) membranes. For each of the samples, three protein blots were prepared. Each blot contained 2.5 µg, 5 µg or 7.5 µg protein for every sample. The membranes were pre-hybridized in 3% skimmed milk (Difco, BD, Franklin Lakes, NJ, USA) in PBS for 1 hr at room temperature, and then were incubated with the primary antibody diluted in TBST buffer (25 mM Tris-HCl at pH 7.4, 150 mM NaCl and 0.1% Tween20) overnight at 4°C. The following primary antibodies were used: acetylated histone H3 (rabbit polyclonal, 1∶20000; Millipore, Billerica, MA, USA) and histone H3 (mouse monoclonal, 1∶200; Millipore). Hybridized bands were visualized using the ECL Plus western blotting detection kit (GE Healthcare, Chalfont St. Giles, UK). The relative densities of the signals were analyzed using an LAS-3000 imaging system (Fujifilm, Tokyo, Japan) and the histone modification levels were compared between samples using the total histone H3 densities as an internal control. Finally, the mean ± SD was calculated from the AcH3 value in the Sus-cultured or TSA-treated samples relative to the controls.

### Statistical Analyses

The MEA tests in DMSO- or TSA-treated CM spheroids were sequentially recorded on Days 2, 6 and 9 for each hESC-CM and hiPSC-CM colony. The average FPD value recorded on Days 6 and 9 was normalized to that of Day 2 (Day 2 = 1) and the relative FPD values were calculated. The changes between Day 2 and Days 6 or 9 were compared to see if there was a difference in the rates of FPD prolongation in the TSA- and DMSO-treated CMs. Prolongation of the relative FPD values would strongly suggest that TSA induced a qualitative shift from immature CMs to mature CMs. In this study, continuous culture of each colony for 9 days on the MEA electrode was necessary. The Mann-Whitney U-test was used, because the sample size was limited and it was not clear if the samples were taken from normally distributed data. For convenience, Z values were used to determine significance in this study. When the absolute value of the calculated Z score was greater than the absolute value of Z critical (1.96), the results were deemed significant (p<0.05). When the absolute value of the calculated Z score was between 1.96 and 1.65, differences were considered a tendency (p<0.1). When the critical values for the U statistic were uses at the 5% level, the difference was significant (p<0.05) only in the TSA- and DMSO-treated hESC-CMs on Day6.

## Supporting Information

Figure S1 **Cardiomyocyte differentiation potencies and function in human iPSC lines.** (A) Comparison of the number of beating colonies derived from five different hiPSC lines to the hESC line, KhES-1 (n = 9). Each graph displays the mean ± SD of three independent experiments. (B) Quantitative RT-PCR (qRT-PCR) analysis of cardiac gene expression in hiPSC-CMs relative to hESC-CMs. Expression levels of β-actin were used as an internal control. Each graph displays the mean ± SD of three independent experiments. (C) Positive effects of 14-day suspension culture (Sus) on cardiac gene expression detected by qRT-PCR analyses in 35-day-old hiPSC-CMs {Sus(d35)} compared with the controls cultured under adhesive conditions {Ad(d35)}. The corresponding values for the human adult hearts (AH) are shown for each sample.
(TIF)

Figure S2 **Microarray data for the expression of genes involved in cardiac ion channel function.** Global gene expression analyses were conducted for TSA- or DMSO-treated hESC-CMs under Ad or Sus culture conditions. Genes involved in sodium, calcium and potassium ion channels were analyzed. The ubiquitously expressed genes, β-actin and RPL13A, were not affected by TSA treatment.
(TIF)

Figure S3 **Transient beating rate increments in response to TSA in hESC/hiPSC-CM colonies.** Beating rates on Day 6 showed a greater reduction on Day 9 after plating in TSA-treated hESC-CMs (A) and hiPSC-CMs (B) compared with DMSO-treated controls. Each bar displays the mean ± SD.
(TIF)

Table S1 Primers for semi-quantitative and quantitative RT-PCR.
(DOC)
