# Supplementary figures and images for: Dynamic Link between Histone H3 Acetylation and an Increase in the Functional Characteristics of Human ESC/iPSC-Derived Cardiomyocytes
Source: PLoS One. 2012 Sep 12;7(9):e45010. doi: 10.1371/journal.pone.0045010 (PMC3440326; doi:10.1371/journal.pone.0045010)

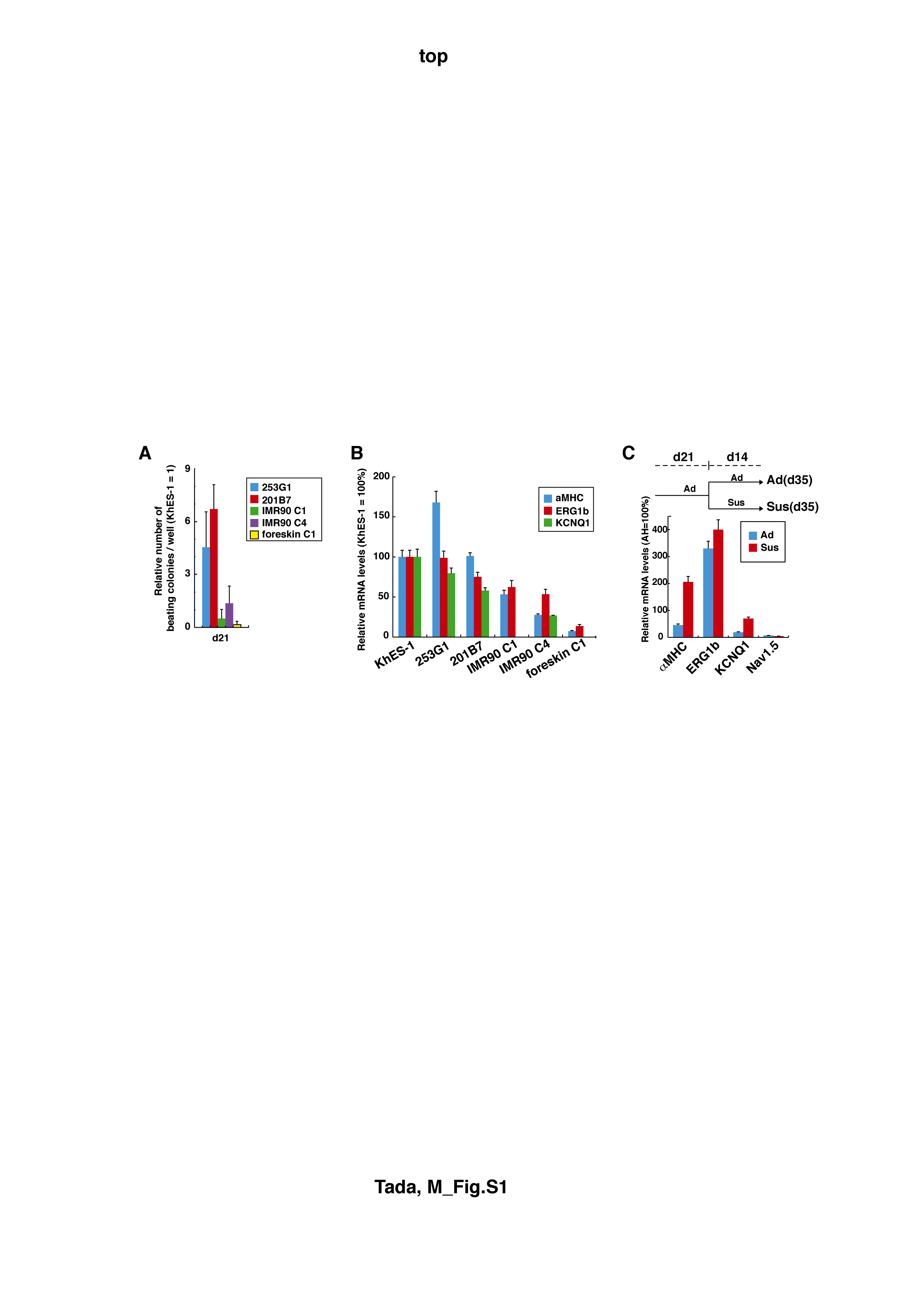

Supplement: Figure S1 — Cardiomyocyte differentiation potencies and function in human iPSC lines. (A) Comparison of the number of beating colonies derived from five different hiPSC lines to the hESC line, KhES-1 (n = 9). Each graph displays the mean ± SD of three independent experiments. (B) Quantitative RT-PCR (qRT-PCR) analysis of cardiac gene expression in hiPSC-CMs relative to hESC-CMs. Expression levels of β-actin were used as an internal control. Each graph displays the mean ± SD of three independent experiments. (C) Positive effects of 14-day suspension culture (Sus) on cardiac gene expression detected by qRT-PCR analyses in 35-day-old hiPSC-CMs {Sus(d35)} compared with the controls cultured under adhesive conditions {Ad(d35)}. The corresponding values for the human adult hearts (AH) are shown for each sample. (TIF) [file pone.0045010.s001.tif]

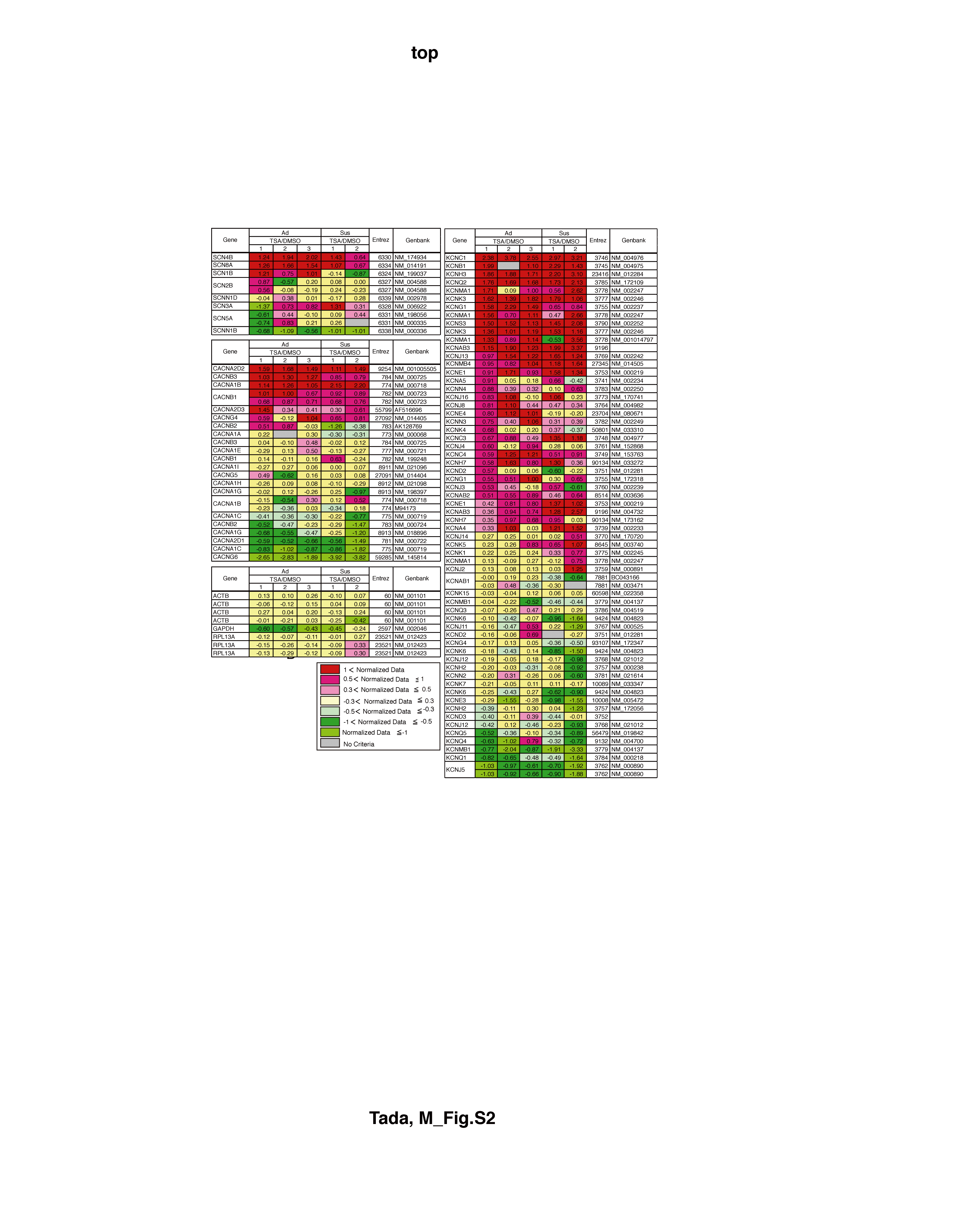

Supplement: Figure S2 — Microarray data for the expression of genes involved in cardiac ion channel function. Global gene expression analyses were conducted for TSA- or DMSO-treated hESC-CMs under Ad or Sus culture conditions. Genes involved in sodium, calcium and potassium ion channels were analyzed. The ubiquitously expressed genes, β-actin and RPL13A, were not affected by TSA treatment. (TIF) [file pone.0045010.s002.tif]

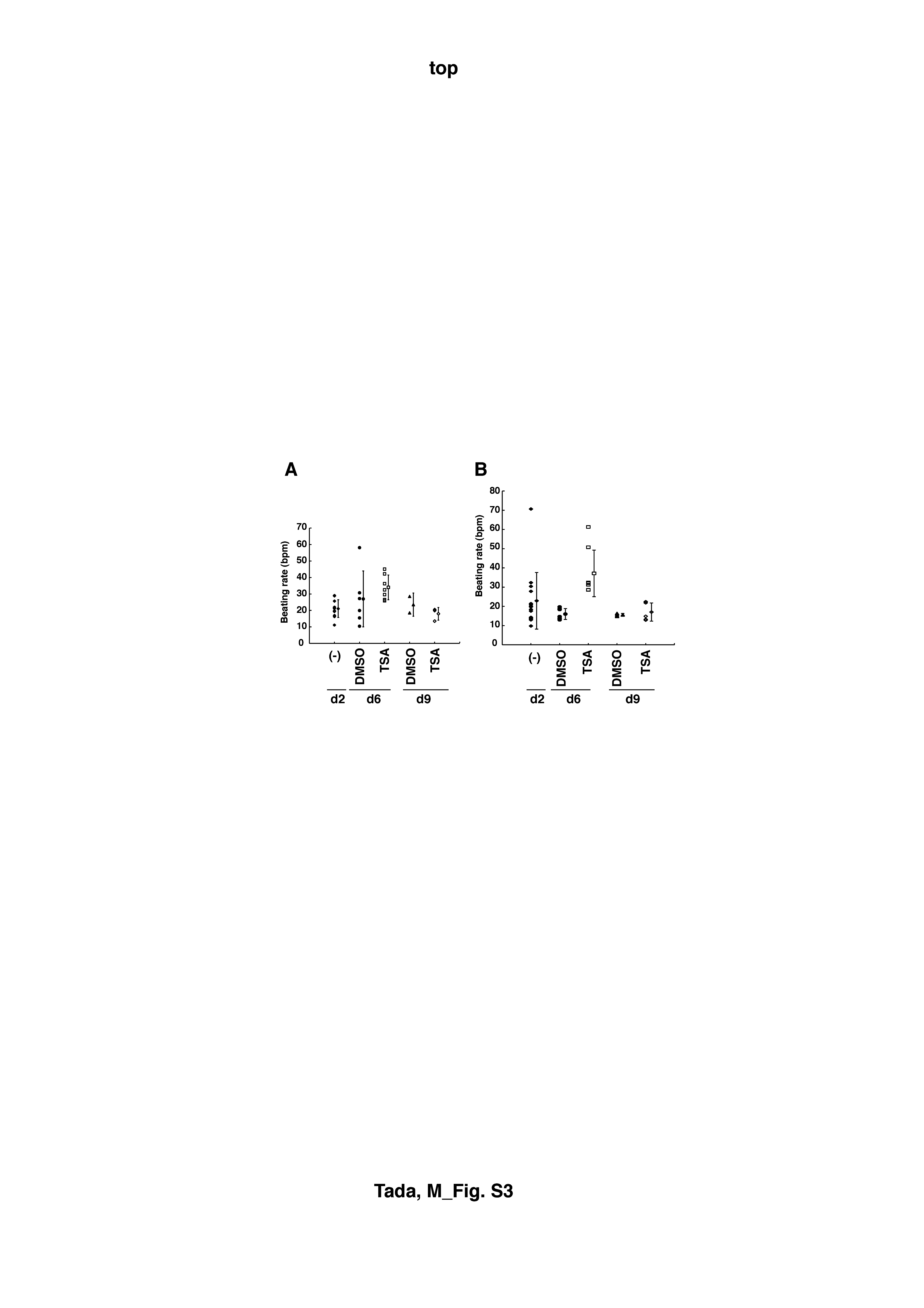

Supplement: Figure S3 — Transient beating rate increments in response to TSA in hESC/hiPSC-CM colonies. Beating rates on Day 6 showed a greater reduction on Day 9 after plating in TSA-treated hESC-CMs (A) and hiPSC-CMs (B) compared with DMSO-treated controls. Each bar displays the mean ± SD. (TIF) [file pone.0045010.s003.tif]
