# Supplementary material for: Dynamic Link between Histone H3 Acetylation and an Increase in the Functional Characteristics of Human ESC/iPSC-Derived Cardiomyocytes
Source: PLoS One. 2012 Sep 12;7(9):e45010. doi: 10.1371/journal.pone.0045010 (PMC3440326; doi:10.1371/journal.pone.0045010)
Supplement: Table S1 — Primers for semi-quantitative and quantitative RT-PCR. (DOC) [file pone.0045010.s004.doc]

**Table S1.** Primers for semi-quantitative and quantitative RT-PCR.

| Gene | Forward primer sequence | Revers primer sequence |
| --- | --- | --- |
| αMHC | ctcaagctcatggccactct | gcctcctttgcttttaccact |
| hERG1b | acgcttactgccagggtgac | gccgactggcaaccagag |
| KCNQ1 | ccacctcaacctcatggtg | acagtgagggcttcccaat |
| Nav1.5 | gagcaacttgtcggtgctg | gatttggccagcttgaagac |
| βACTIN | attggcaatgagcggttc | ggatgccacaggactccat |
